# Morphology and Mechanical Properties of Fossil Diatom Frustules from Genera of *Ellerbeckia* and *Melosira*

**DOI:** 10.3390/nano11061615

**Published:** 2021-06-20

**Authors:** Qiong Li, Jürgen Gluch, Zhongquan Liao, Juliane Posseckardt, André Clausner, Magdalena Łępicka, Małgorzata Grądzka-Dahlke, Ehrenfried Zschech

**Affiliations:** 1 Fraunhofer Institute for Ceramic Technologies and Systems IKTS, Maria-Reiche-Str. 2, 01109 Dresden, Germany; juliane.posseckardt@ikts.fraunhofer.de (J.P.); andre.clausner@ikts.fraunhofer.de (A.C.); ehrenfried.zschech@ikts.fraunhofer.de (E.Z.); 2 Institute of Physics, Faculty 1, Brandenburg University of Technology Cottbus-Senftenberg, Konrad-Zuse-Str. 1, 03044 Cottbus, Germany; 3 Institute of Mechanical Engineering, Faculty of Mechanical Engineering, Bialystok University of Technology, Wiejska Str. 45C, 15-531 Bialystok, Poland; m.lepicka@pb.edu.pl (M.Ł.); m.dahlke@pb.edu.pl (M.G.-D.)

**Keywords:** diatom, fossil frustule, 3D visualization, X-ray computed tomography, micromechanical behavior, morphology

## Abstract

Fossil frustules of *Ellerbeckia* and *Melosira* were studied using laboratory-based nano X-ray tomography (nano-XCT), transmission electron microscopy (TEM) and energy-dispersive X-ray spectroscopy (EDS). Three-dimensional (3D) morphology characterization using nondestructive nano-XCT reveals the continuous connection of fultoportulae, tube processes and protrusions. The study confirms that *Ellerbeckia* is different from *Melosira*. Both genera reveal heavily silicified frustules with valve faces linking together and forming cylindrical chains. For this cylindrical architecture of both genera, valve face thickness, mantle wall thickness and copulae thickness change with the cylindrical diameter. Furthermore, EDS reveals that these fossil frustules contain Si and O only, with no other elements in the percentage concentration range. Nanopores with a diameter of approximately 15 nm were detected inside the biosilica of both genera using TEM. In situ micromechanical experiments with uniaxial loading were carried out within the nano-XCT on these fossil frustules to determine the maximal loading force under compression and to describe the fracture behavior. The fracture force of both genera is correlated to the dimension of the fossil frustules. The results from in situ mechanical tests show that the crack initiation starts either at very thin features or at linking structures of the frustules.

## 1. Introduction

Natural materials are evolutionary optimized materials that frequently feature hierarchical structures. They play an important role in bionanotechnology and composite materials [1,2,3,4,5,6,7]. Diatom biosilica is one of these biological materials, mainly from two sources: diatomaceous earth and living diatoms. Diatoms are unicellular microorganisms with more than hundreds of thousands of species [8]. Their hard and porous wall with a unique 3D morphology is called diatom frustule [9,10,11]. Biosilica from diatom frustules resist disintegration and decay, and therefore, diatom-rich fossil deposits have formed over time at many sites (e.g., oceans, lakes and marshes) [12,13]. This naturally formed diatomaceous earth (or diatomite) is an abundant mineral source and distributes widely on earth [14,15], by which it can be easily obtained at a large scale and at a low cost. During the time of forming fossil frustules (diatomaceous earth), there could be other elements incorporated into the biosilica that become part of the chemical composition. Elemental analysis showed the diatomaceous earth contains a high amount of oxygen (48 wt. %) and silicon (46 wt. %) with a small amount of aluminum (3 wt. %) and iron (3 wt. %), as well as other elements [16]. However, the chemical composition of diatomaceous earth varies from region to region [17,18].

Because fossil frustules are lightweight and their specific mechanical properties have been optimized during evolution [19,20], they could be used as components in composites. So far, the market of lightweight and high-strength composite materials is growing fast [21]. Many metal matrix composites use ceramic particles such as Al_2_O_3_, SiC, MO, B_4_C, and TiC as reinforcing components. Only limited studies exist for SiO_2_-based composites [22,23,24,25]. To date, few studies have been conducted on the use of fossil frustules as reinforcement components in metal matrix composites (MMCs). Before using fossil frustules as functional materials in MMCs, the morphology and mechanical properties of the selected fossil frustules must be known. However, because the size of most diatom species ranges from 3 µm to 200 µm [26], traditional mechanical test methods struggle to characterize individual frustules. Recently, new approaches on micro- and nanomechanical tests have been developed and used to study the mechanical behavior of frustules [20,27,28,29]. Indentation by atomic force microscopy (AFM) on pennate and centric diatoms showed that the elastic modulus varied from several to hundreds GPa depending on the test locations [27,28]. In situ microindentation, in combination with scanning electron microscopy (SEM) on pennate diatom frustules (*Didymosphenia geminata*), shows that the applied load to achieve elastic deformation on the epivalve surface ranges from 100 µN to 1000 µN [29]. In three-point bending experiments on the centric diatom (*Coscinodiscus* sp.), fracture was observed at an average stress of 1.1 GPa [20]. So far, no studies on the mechanical behavior of a whole fossil frustule under compressive load using a flat punch have been published. Such experiments will provide an understanding of the fracture behavior of individual frustules under compression. Only the study from Hamm et al. [30] using glass microneedles to load and break three living species revealed that the force required to break the living diatom is in the range from dozens to hundreds of µN depending on the diameter of diatoms. Furthermore, one of the most critical points for the application of fossil frustules in MMCs is to understand the exact intricate 3D morphologies of the frustules. SEM, also in combination with a focused ion beam (FIB), and AFM, were used to study the morphologies of the diatom frustules [9,10,28,29,31,32]. However, these techniques cannot access the interior structure nondestructively. To achieve the 3D morphology of the fossil frustules and to correlate the structure to the mechanical properties, a combination of nondestructive 3D imaging with in situ micromechanical tests is required.

In this study, the studied fossil frustules from diatomaceous earth belong to the genera of *Ellerbeckia* and *Melosira*. All of them are centric diatoms with cylindrical shapes and feature a diameter in the range from 40 µm to 80 µm. Nano-XCT is used to image these frustules and to provide data to reconstruct their 3D structures. Furthermore, in situ mechanical compression tests are combined with nano-XCT imaging to investigate the maximum loading force for the whole fossil frustule and the fracture behavior of individual fossil frustules. To obtain the nanostructure and the chemical composition of the fossil frustules, thin lamellae were prepared by FIB and studied by TEM.

## 2. Materials and Methods

### 2.1. Morphology and Chemical Composition Study

Fossil diatom frustules were taken from diatomaceous earth (Perma-Guard Inc., North Salt Lake, UT, USA, Batch No. 3/9/17305). This diatomaceous earth has a large fraction of complete frustules. The particle density of the fossil frustules in diatomaceous earth determined by the helium pycnometry technique is about 2.23 g/cm^3^. The bulk density measured by the Hall flowmeter with an aperture of 2.5 mm in diameter is about 0.19 g/cm^3^. Complete frustules of *Ellerbeckia* and *Melosira* were selected for this study. For nano-XCT (Xradia nano-XCT-100, photon energy = 8 keV, Xradia Inc., Pleasanton, CA, USA) experiments, an intact frustule was selected and fixed on the tip of a sample holder. A gold fiducial marker was carefully positioned on top of the frustule for alignment during the tomography and tomographic reconstruction. Individual images were acquired in Zernike phase-contrast mode [33]. The detailed experimental setup of the nano-XCT tool was described in [34]. All imaging was performed in a large field of view of 66.5 × 66.5 µm^2^ with 512 × 512 pixels per image. The complete tomographic data sets consist of 601 images, collected over 180° (parallel beam geometry) with an exposure time of 230 s per image. The images were aligned using a custom plugin in ImageJ [35] and subsequently reconstructed using the Xradia Inc. (Pleasanton, CA, USA) commercial software package (Xradia XMReconstructor).

For chemical composition analysis and nanostructure characterization of fossil *Ellerbeckia* and *Melosira* frustules, TEM lamellae were prepared using a Dual Beam SEM-FIB system (NVision 40, Carl Zeiss AG, Oberkochen, Germany) and imaged using a scanning TEM (Libra 200 MC Cs, Carl Zeiss AG, Oberkochen, Germany) at an accelerating voltage of 200 kV. Element mapping of the lamellae was performed using energy-dispersive X-ray spectroscopy (EDS) in the TEM.

### 2.2. Micromechanical Behavior Study

In situ micromechanical compression tests were performed inside the nano-XCT system with an in-house-developed micromechanical test setup [36]. For the mechanical testing, a single fossil frustule from genera of *Ellerbeckia* and *Melosira* was selected and compressed between two flat surfaces (bottom: steel sample holder, top: diamond flat punch) by stepwise increasing loads. The loading force was determined and a radiograph with a 60 s exposure time was recorded at each step. Tomographies were acquired before and after the compression test. The full tomography before the indentation test was used to verify the integrity of the sample. The tomography data acquired after the indentation test were to evaluate the 3D crack pattern. All tomographies of the samples from mechanical tests consist of 401 images collected during 180° rotation with an exposure time of 120 s for each image.

## 3. Results and Discussion

### 3.1. Morphology and Chemical Composition Study

**Figure 1:**
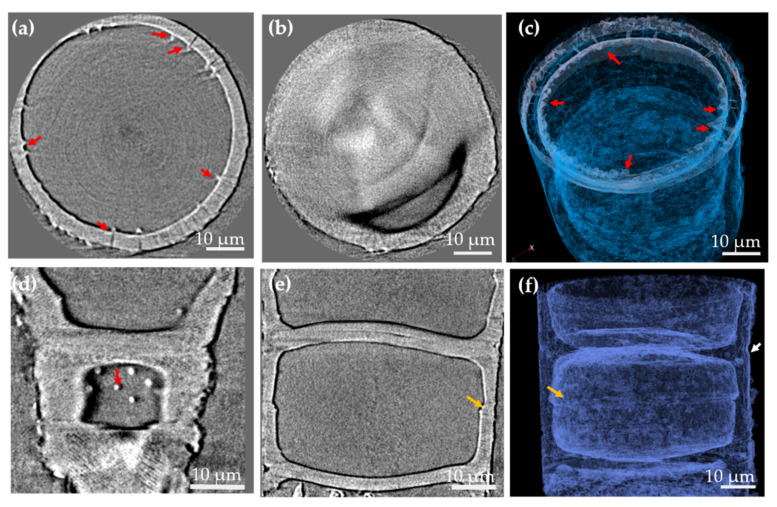
*Ellerbeckia* frustule studied by nano-XCT. (**a**,**b**) Valve view of the frustule, (**d**,**e**) girdle view of the frustule and (**c**,**f**) 3D rendering of *Ellerbeckia* in valve view and girdle view, respectively. Red arrows: protrusions, white arrow: pseudosulcus, orange arrows: the region linking the epivalve and hypovalve.

**Figure 2:**
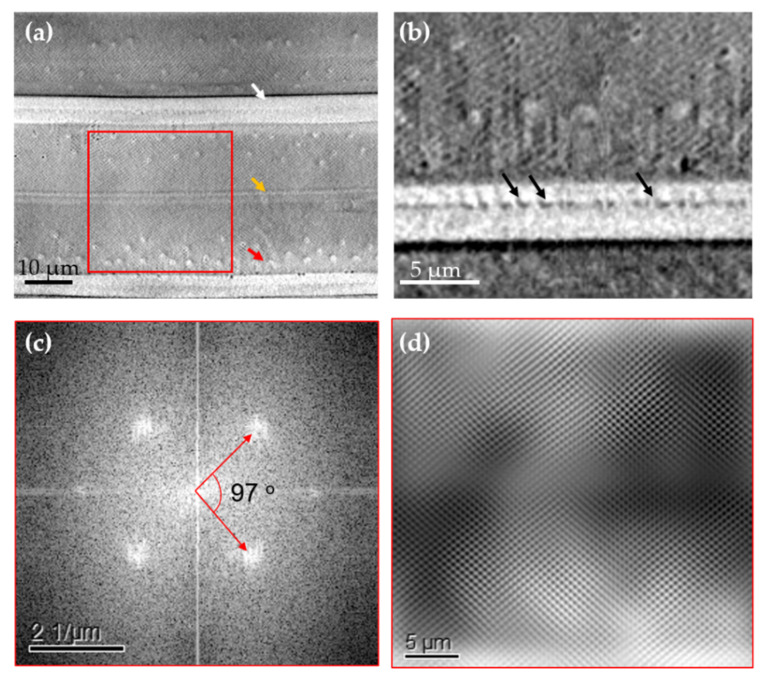
Data analysis of the *Ellerbeckia* mantle wall. (**a**) Part of the unfolded frustule wall, (**b**) magnified imaging of the morphology of the interlocking series of ridges and grooves (black arrows) on the valve faces, (**c**) FFT analysis of pores on the frustule wall from the red square area in (**a**,**d**) reconstructed outer wall.

Nano-XCT data of a fossil frustule from *Ellerbeckia* with a cylindrical shape and a diameter of about 60 µm are shown in Figure 1. It is found from reconstructed 3D data of the *Ellerbeckia* frustule that there are fultoportulae on the surface of the mantle wall, crossing through the mantle wall and forming protrusions inside the frustule wall (Figure 1a,c,d and Figure 2a). Light microscopy (LM) and SEM images show the morphology of the protrusions on the surface wall (Figure S1a) and at the inside of the frustule wall (Figure S1b,c). Furthermore, the 3D rendering of the frustule in girdle view (Figure 1f) shows a pseudosulcus (white arrow) at the rims of the two valve faces and a linking region of the epivalve and hypovalve (orange arrow). By virtually unrolling the cylinder wall of *Ellerbeckia* (Figure 2a), a flat view of the cylindrical wall shows the external openings of fultoportulae (red arrow), the linking region of the valve faces (white arrow) and the region linking the epivalve and hypovalve (orange arrow). With a higher magnification of the linking region of the valve faces, interlocking series of ridges and grooves (black arrows) on the valve are observed (Figure 2b). Applying fast Fourier transform (FFT) analysis on the unrolled wall (red square area in Figure 2a), pores arranged in a periodic rhombus geometry with around an 83° angle are revealed for this specific *Ellerbeckia* frustule (cylindrical diameter: 63 µm). However, the frustule with a cylindrical diameter of 60 µm shows an angle of 71° (Figure S2). These data demonstrate the wide variability of the structural parameters of the frustules [32]. The average distance between two pores is about 0.6 µm, and the pore size is about 0.1 µm in diameter.

**Figure 3:**
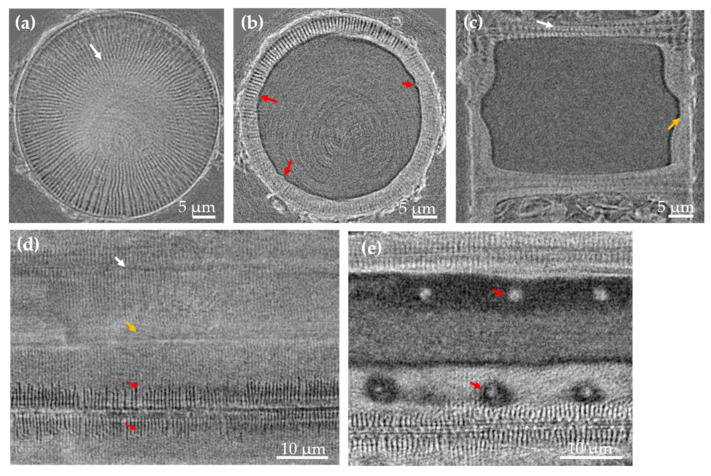
Nano-XCT images of the *Melosira* frustule. (**a**,**b**) Valve view of the frustule and (**c**) girdle view of the frustule, unfolded cylindrical wall of the *Melosira* frustule from the surface (**d**) to the interior (**e**).

Nano-XCT images of a *Melosira* frustule with a cylindrical diameter of about 45 µm are shown in Figure 3. Radial striae are found on the valve faces (Figure 3a, white arrow), and tube processes (Figure 3b, red arrows) are distributed through the mantle wall. *Melosira* in girdle view (Figure 3c) shows the corresponding morphology of the two linked valve faces (white arrow) and a linking region of the epivalve and hypovalve (orange arrow). Figure 3d again shows the flat view of the unrolled cylindrical surface wall with distinguished striae extending from the valve face to the mantle area (red arrows), the linking region of the valve faces (white arrow) and the region to link the epivalve and hypovalve (orange arrow). However, the pores here do not form a pattern as in the frustules of *Ellerbeckia* (Figure 2 and Figure S3). Exploring from the surface to the interior of the unrolled wall, a few protrusions (red arrows in Figure 3e) at the inside of the frustule wall are visible.

The study of Crawford and Sims [37] shows the species from the genus of *Ellerbeckia* are different from the species from the genus of *Melosira.* Although the species from the genus of *Ellerbeckia* are still under the name of genus of *Melosira* in the Algaebase database [38,39,40], the morphological features derived from Figure 1, Figure 2 and Figure 3 specifically show that *Ellerbeckia* is a different genus than *Melosira*. For example, between the two frustules of *Ellerbeckia*, there is a pronounced pseudosulcus (Figure 1f) and an interlocking series of ridges and grooves on the outer rim of the valve faces (Figure 2b), while for the *Melosira* frustules, there are special structures to link the two valve faces together (Figure 3c) with no pseudosulcus. The mantle wall of *Ellerbeckia* is evenly thick with external openings of fultoportulae and sharp protrusions inside (red arrows in Figure 1), while the mantle wall of *Melosira* is unevenly thick with blunt and fewer protrusions at the inside wall (red arrows in Figure 3b,e). Although there are different morphologies, the images of both the *Ellerbeckia* and *Melosira* frustules show typical heavily silicified frustules with thick frustule walls. Moreover, both genera form a cylindrical shape with the valve faces linking together to form chains (Figure 1e,f, Figure 3c, Figures S1a and S5a) [37,38,39,40,41]. Tube processes are present in the mantle wall of these frustules. (Figure 1a,c and Figure 3b), and they form protrusions at the inside wall (Figure 1d and Figure 3e). However, due to the limited resolution, no information about the nanostructure inside the biosilica is provided by nano-XCT imaging. Therefore, thin lamellae of three different fossil frustules were prepared by FIB and imaged by TEM: (i) valvocopula, (ii) linking region of the two valve faces and (iii) the center of the valve face. Additionally, EDS mapping was performed to analyze the chemical composition of these fossil frustules.

**Figure 4:**
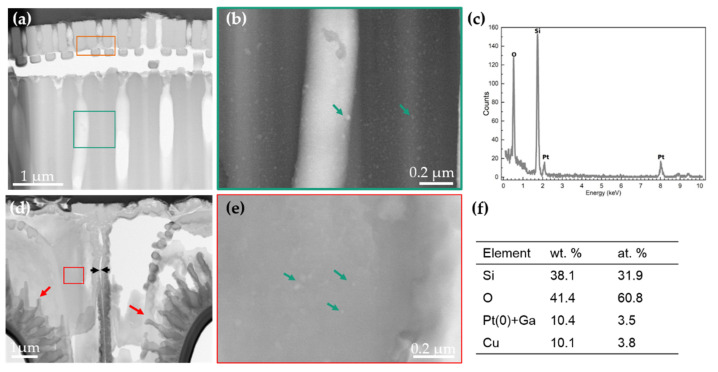
TEM imaging and chemical composition analysis of *Ellerbeckia* (**a**–**c**) in the area of the valvocopula and *Melosira* (**d**–**f**) in the linking region of the two valve faces. (**a**) Image of the region of the valvocopula, (**b**) image of the green area in (**a**) at a higher magnification, (**c**) spectrum of elements in the orange area of (**a**), (**d**) image of the linking region of the two valve faces, (**e**) high-magnification image of the red area in (**d**) and (**f**) area spectrum of elements in the red area. Green arrows: nanopores; red arrows: tube processes; black arrows: a special structure to link the two valve faces. Pt and Ga content arises from the FIB preparation, and Cu is from the sample holder.

Figure 4a shows the TEM images of the valvocopula of *Ellerbeckia*. The substructure of this region is shown in Figure 4b. In that area, nanopores with a diameter of about 15 nm are found (Figure 4b). EDX (Figure 4c) shows that the frustule in this region contains only O and Si, i.e., SiO_2_. (Figure S4c). TEM imaging of *Melosira* in the linking region of the two valve faces shows tube processes under the radial striae of the valve surface (Figure 4d, red arrows) and a special structure (Figure 4d, black arrows) to link the two valve faces, also shown in Figure 3c (white arrow) by nano-XCT imaging. Nanopores with a diameter of about 15 nm are also found in this area (Figure 4e, green arrows). Composition analysis of the red area in Figure 4d shows again that the frustule material of the investigated sample contains pure Si and O (Figure 4f). The valve face center of *Ellerbeckia* also has nanopores with a diameter of about 15 nm and has the same chemical composition (Figure S6). The EDS study in the TEM shows only Si and O without other elements (or beyond the detection limit of about 0.1 wt. %) in fossil frustules, which proves that the source is very pure, similar to the frustules from the cultured diatoms [20,29].

### 3.2. Uniaxial Static Compression Test of Fossil Frustules

**Figure 5:**
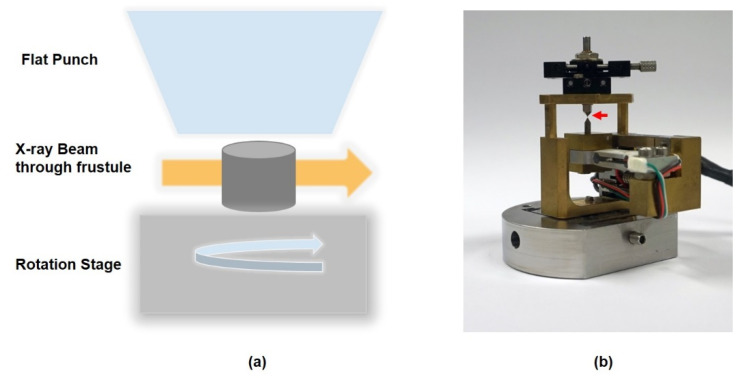
In situ compression test in nano-XCT. (**a**) Schematic diagram of the compression loading on the frustule inside the nano-XCT system and (**b**) photo of the compression loading sample stage, red arrow: pin with sample.

In situ uniaxial compression tests (Figure 5) were performed by a nanomechanical test device integrated into nano-XCT. More details of the setup are provided in [36,42]. As shown in Figure 5a, the whole compression setup allows acquiring radiographs from a large tilt range for limited-angle tomography. The compression setup consists of a piezomechanical actuator, a force gauge and two anvils. In this study, a diamond flat punch was used as the upper anvil.

**Figure 6:**
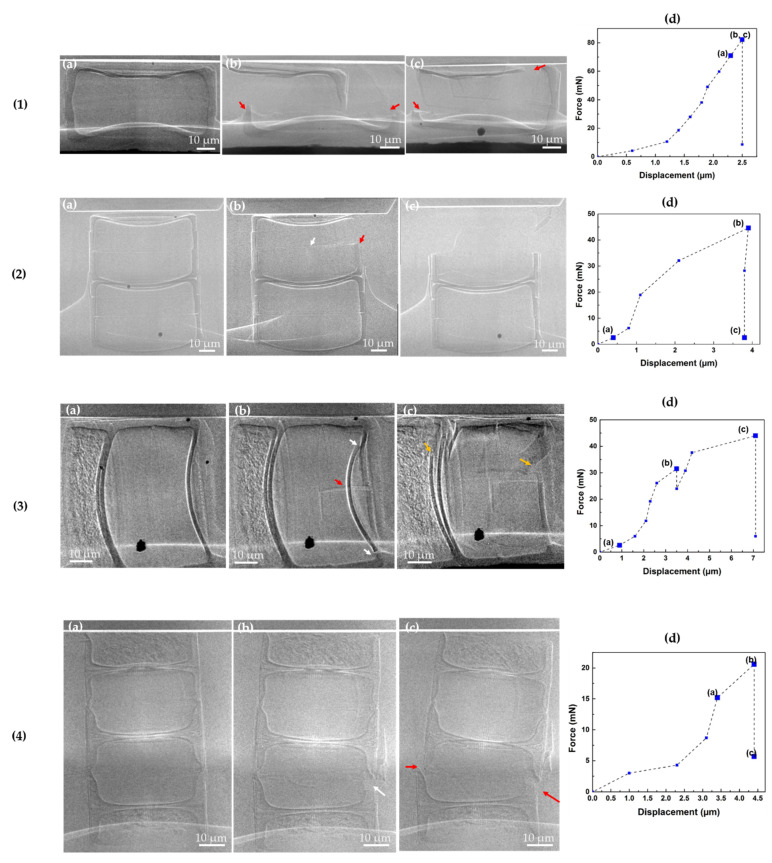
In situ mechanical compression tests on studying the mechanical behavior of the four different fossil frustules (**1**–**4**). (**a**–**c**) Radiographs recorded during the mechanical test and (**d**) corresponding load-displacement curves. The enlarged data points in (d) correspond to the radiographs in (**a**–**c**). Dashed lines are a guide for the eye.

The compression tests were carried out on either the whole valve face or on the copulae area to determine the maximal loading force (F_max_) and to understand the crack initiation of these individual fossil frustules (Figure 6, Table 1). Figure 6(1), (2) and (4) represent the compression test on the valve face for *Ellerbeckia* frustules and the *Melosira* frustule, with cylindrical diameters of 80 µm, 69.5 µm and 46 µm, respectively. Figure 6a of these frustules shows the samples under load before any cracks happen. Figure 6b,c of the *Ellerbeckia* frustule Figure 6(1) are two views from perpendicular directions of the broken frustule. The crack of these three frustules started from the linking region of the epivalve and hypovalve (Figure 6b, white arrows), and their maximum loading forces under compression are 82.2 mN, 45.2 mN and 20.6 mN, respectively. For the *Ellerbeckia* frustule Figure 6(2), a part of the hypovalve (Figure 6(2)b, red arrow) started to break right after the crack initiation. It then cracked further, and the whole hypovalve shattered (Figure 6(2)c). For the *Melosira* frustule Figure 6(4), a total collapse (Figure 6(4)c, red arrows) took place at the initial crack region under F_max_.

Figure 6(3) shows the compression test on the copulae area of an *Ellerbeckia* frustule with a cylindrical diameter of 62.5 µm. Here, the frustule started to crack at the right side in the valve face (Figure 6b, white arrows), and a small collapse took place here (Figure 6(3)d). As force increased, a large crack was observed (Figure 6(3)b, red arrow), and then the whole diatom shattered from the valve faces of both sides (Figure 6(3)c, orange arrows) under the maximum loading force of 44.1 mN (Figure 6(3)d, Table 1).

**Figure 7:**
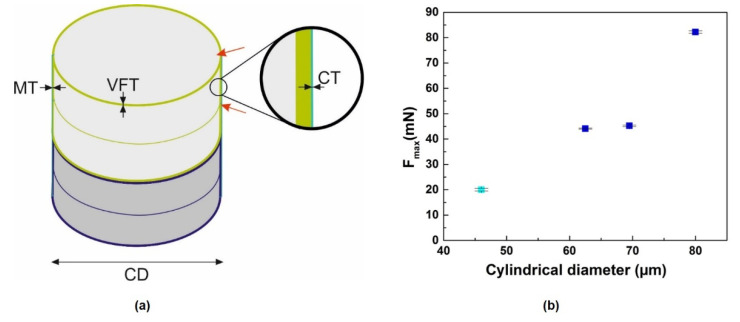
Diagrams of the studied fossil frustules. (**a**) Schematic diagram of studied fossil frustules with two diatom cells (light green and blue cylinders). Indicated are the valve mantle thickness (MT), valve face thickness (VFT), copulae thickness (CT) and cylindrical diameter (CD). Red arrows: positions of crack initiation. (**b**) Graph of the tendency of the maximum loading force on the cylindrical diameter. Blue points: *Ellerbeckia*, cyan point: *Melosira*.

The results from compression tests (Figure 6 and Figure 7b) show that the maximal force is related to the cylindrical diameter of the frustules. For these specific fossil frustules from genera of *Ellerbeckia* and *Melosira*, F_max_ decreases as the structure geometry of these frustules decreases (Table 1, Figure 7b). Hamm et al. [30] revealed that loading force and size were inversely related not only within the same species of *Thalassiosira punctigera* but also between different genera (*Coscinodiscus* and *Fragilariopsis*). However, the reason behind the loading force–size tendency was not clearly stated in that study. In order to understand why the maximal loading force decreases as the cylindrical diameter of the fossil frustules decreases, the structural parameters of valve thickness, mantle wall thickness and copulae thickness from 3D reconstruction data were measured and compared (Figure 7a, Table 1 and Table 2). The absolute values of valve face thickness and mantle wall thickness from both genera decrease as the cylindrical diameter decreases. However, the ratio of valve face thickness (VFT), mantle thickness (MT) and copulae thickness (CT) to cylindrical diameter (CD) for *Ellerbeckia* remains constant despite the different diameters.

The radiographs recorded during the compression tests (Figure 6) show that the crack initiates from either the linking region of the epivalve and hypovalve or from the valve face. Average values for the ratios of VFT, MT and CT to CD from *Ellerbeckia* are 0.029 ± 0.002, 0.042 ± 0.002, and 0.010 ± 0.001, respectively. Among these ratios, MT/CD is larger than VFT/CD and CT/CD, i.e., the weakest points for *Ellerbeckia* are the valve face and copulae. Figure 6(1–3) shows that the crack starts from the valve face or the linking region of the epivalve and hypovalve, i.e., the copulae. For *Melosira* (i.e., Frustule 4) with a similar cylindrical architecture compared to *Ellerbeckia*, the absolute values of valve face thickness and mantle wall thickness are smaller (Table 1 and Table 2). Moreover, VFT/CD increases from 0.029 to 0.035, CT/CD increases from 0.010 to 0.017, while the MT/CD ratio decreases from 0.042 to 0.025. That means, the weak point for *Melosira* is the region of the mantle wall, i.e., the linking region of the epivalve and hypovalve (Figure 3c, orange arrow). Figure 6(4)b shows that the crack starts exactly from that point (white arrow).

The observed maximum loading force in this study is hundreds of times higher than in the study of Hamm et al. [30]. The reason could be that *Ellerbeckia* and *Melosira* are heavily silicified frustules with valve faces linking together to form cylindrical chains, so the 3D morphology is different from *Thalassiosira*. *Thalassiosira* is not heavily silicified and does not form a cylindrical chain [43,44]. Additionally, the compression testing here is on fossil frustules, while Hamm et al. performed compression tests on living diatoms. Furthermore, the analysis of 3D reconstruction data of *Thalassiosira lacustris* in Figure S7 and Table S1, the same genus as *Thalassiosira punctigera* in the study of Hamm et al., reveals the absolute values of valve thickness, mantle wall thickness and copulae thickness do not increase as the valve diameter increases. These values remain almost constant as the valve diameter decreases.

## 4. Conclusions

The 3D morphology of fossils, centric frustules (genera: *Ellerbeckia* and *Melosira*), was examined using nano-XCT. It was verified that *Ellerbeckia* is a different genus than *Melosira.* The studied *Ellerbeckia* frustules lack striae on the valve faces and feature a pseudosulcus at the rim between two frustules. The mantle wall is evenly thick with pores in rhombic arrangement on the frustule wall. For *Melosira* frustules, the valve faces are linked by special substructures. The mantle wall is unevenly thick with the thinnest structure on the linking region of the epivalve and hypovalve. Additionally, there are fewer blunt protrusions inside the mantle wall of the *Melosira* frustules. However, the 3D morphology of both *Ellerbeckia* and *Melosira* frustules is cylindrical in shape, and the diatom cells are connected to each other by their valve faces. EDS studies confirm that fossil frustules of both genera contain Si and O only, with no other elements with a concentration above the detection limit of 0.1 wt. %. TEM imaging reveals nanopores with a size of about 15 nm distributed almost everywhere inside the biosilica.

In situ micromechanical experiments with uniaxial loading were carried out within a nano-XCT tool to determine the mechanical properties of the individual fossil frustule. For these heavily silicified fossil frustules, with similar cylindrical architecture and chemical composition, the maximum loading force is related to the dimension of the fossil frustule. Analysis of the morphological parameters shows that the absolute values of VFT, MT and CT decrease as the cylindrical diameter decreases for both genera, except for the CT of *Melosira*. The average values of the ratios VFT/CD, CT/CD and MT/CD and the results from mechanical compression tests indicate the valve face, as well as the linking region of the epivalve and hypovalve as weak spots. Together with the compression force data, a relationship between the maximum applicable force inducing crack initiation and the size of the fossil frustules is provided. This information might enlighten the studies when using the fossil frustules for functional materials design, e.g., as a filler material in lightweight metal matrix composites.

## Figures and Tables

**Table 1:** Structure parameters and maximal loading force (F_max_) of the frustules in mechanical tests.

Sample	F_max_	Cylindrical Diameter (CD)	Valve Thickness (VFT)	Mantle Wall Thickness (MT)	Copulae Thickness (CT)
	mN	µm	µm	µm	µm
Frustule 1 (*Ellerbeckia*)	82.2 ± 0.5	80.0 ± 0.9	2.43 ± 0.1	3.5 ± 0.2	0.81 ± 0.07
Frustule 2 (*Ellerbeckia*)	45.2 ± 0.3	69.5 ± 0.7	1.99 ± 0.07	2.9 ± 0.3	0.75 ± 0.03
Frustule 3 (*Ellerbeckia*)	44.1 ± 0.2	62.5 ± 0.7	1.71 ± 0.08	2.6 ± 0.2	0.67 ± 0.02
Frustule 4 (*Melosira*)	20.6 ± 0.5	46.0 ± 0.4	1.6 ± 0.3	1.16 ± 0.09 *	0.78 ± 0.08

* Because of the uneven mantle wall of the *Melosira*, the data are the average value from the linking region of the epivalve and hypovalve on the mantle wall (orange arrow in Figure 3c).

**Table 2:** Comparison of the different structure parameters with the cylindrical diameter.

Sample	VFT/CD	MT/CD	CT/CD
Frustule 1 (*Ellerbeckia)*	0.030	0.044	0.010
Frustule 2 (*Ellerbeckia)*	0.029	0.041	0.011
Frustule 3 (*Ellerbeckia)*	0.027	0.041	0.011
Frustule 4 (*Melosira)*	0.035	0.025	0.017

## Data Availability

The data presented in this study are available on request from the corresponding author. This data are not publicly available due to the excessive size and complex format.

## Supplementary Materials

The following are available online at https://www.mdpi.com/article/10.3390/nano11061615/s1, Figure S1: Light microscopy (LM) and SEM imaging of the *Ellerbeckia* frustules, Figure S2: Data analysis of the *Ellerbeckia* mantle wall with a cylindrical diameter of 60 µm, Figure S3: FFT analysis of the unrolled surface wall of the *Melosira* frustule, Figure S4: Chemical composition analysis of *Ellerbeckia* in the area of the valvocopula, Figure S5: LM and SEM imaging of the *Melosira* frustule, Figure S6: TEM imaging and EDX of *Ellerbeckia* in the center of the intaglio valve face, Figure S7: Nano-XCT images of *Thalassiosira lacustris*, Table S1: Structure parameters of the frustules from *Thalassiosira lacustris*.
