# Supplementary material for: Morphology and Mechanical Properties of Fossil Diatom Frustules from Genera of Ellerbeckia and Melosira
Source: Nanomaterials (Basel). 2021 Jun 20;11(6):1615. doi: 10.3390/nano11061615 (PMC8235678; doi:10.3390/nano11061615)
Supplement: Supplementary file 1 [file nanomaterials-11-01615-s001.zip › nanomaterials-1233302-supplementary.pdf]

Supplementary Materials

Morphology and mechanical properties of fossil diatom frustules from genera of *Ellerbeckia* and *Melosira*

Qiong Li [1,2,*], Jürgen Gluch [1,*], Zhongquan Liao [1,*], Juliane Posseckardt [1], André Clausner [1], Magdalena Łępicka [3], Małgorzata Grądzka-Dahlke [3] and Ehrenfried Zschech [1,2]

1 Fraunhofer Institute for Ceramic Technologies and Systems IKTS, Maria-Reiche-Str. 2, 01109 Dresden, Germany; qiong.li@ikts.fraunhofer.de (Q.L.); juergen.gluch@ikts.fraunhofer.de (J.G.); zhongquan.liao@ikts.fraunhofer.de (Z.L.); juliane.posseckardt@ikts.fraunhofer.de (J.P.); andre.clausner@ikts.fraunhofer.de (A.C.); ehrenfried.zschech@ikts.fraunhofer.de (E.Z.)

2 Institute of Physics, Faculty 1, Brandenburg University of Technology Cottbus-Senftenberg, Konrad-Zuse-Str. 1, 03044 Cottbus, Germany.

3 Institute of Mechanical Engineering, Faculty of Mechanical Engineering, Bialystok University of Technology, Wiejska str. 45C, 15-531 Bialystok, Poland; m.lepicka@pb.edu.pl (M.L); m.dahlke@pb.edu.pl (M.G-D.)

* Correspondence: qiong.li@ikts.fraunhofer.de (Q.L.); juergen.gluch@ikts.fraunhofer.de (J.G.) zhongquan.liao@ikts.fraunhofer.de (Z.L.)

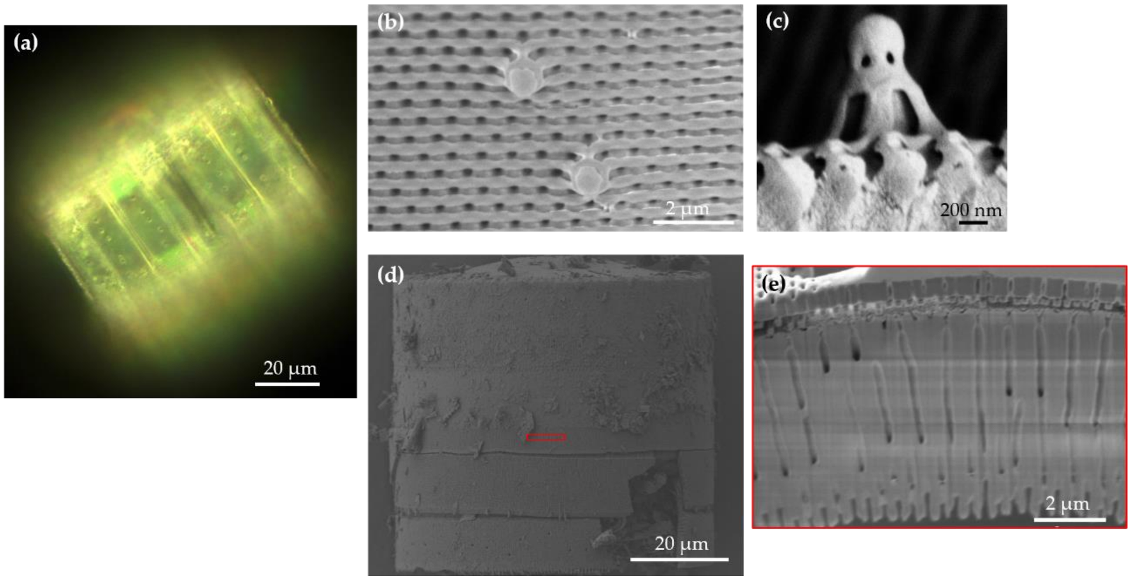

Figure S1. Light microscopy (LM) and SEM imaging of the *Ellerbeckia* frustules. (a) LM imaging of the *Ellerbeckia* frustule with two diatoms linking to form chain, (b) SEM imaging of the protrusions from a broken *Ellerbeckia* frustule, the protrusions located inside the frustule wall, (c) high magnification side view of one protrusion, (d) SEM imaging of the *Ellerbeckia* frustule (a) prepared for the TEM lamella (e).

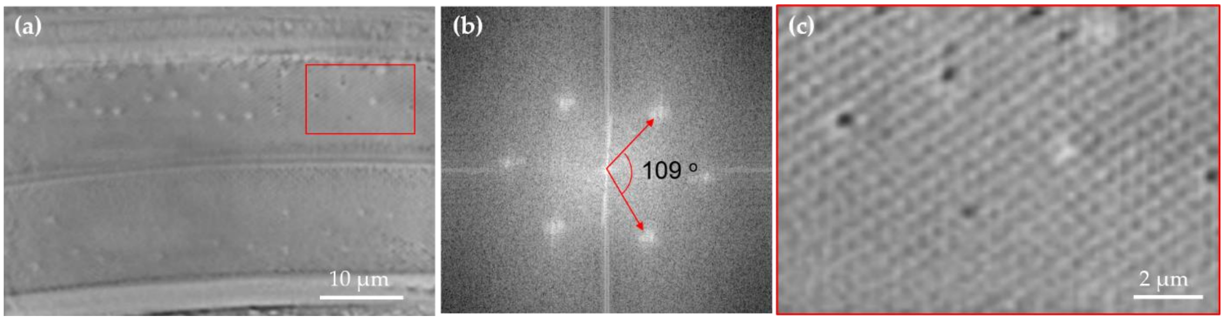

Figure S2. Data analysis of *Ellerbeckia* mantle wall with cylindrical diameter of 60 µm. (a) Part of the unfolded frustule wall, (b) FFT analysis of pores on the frustule wall, (c) the magnified imaging of the red area in (a).

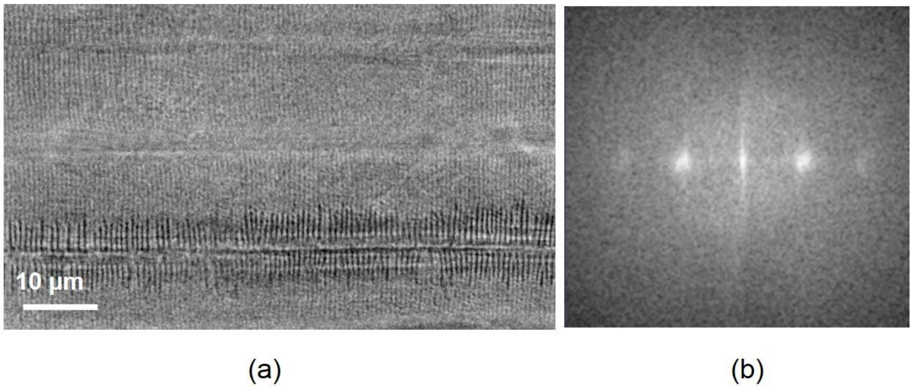

Figure S3. FFT analysis the unrolled surface wall of *Melosira* frustule.

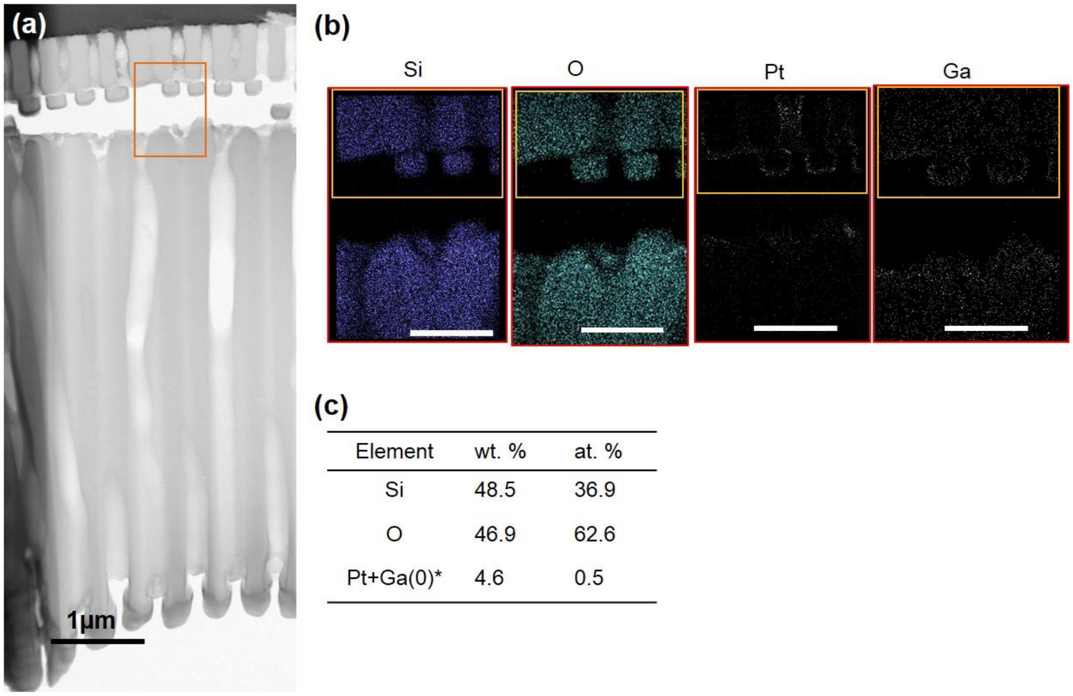

Element	wt. %	at. %
Si	48.5	36.9
O	46.9	62.6
Pt+Ga(0)*	4.6	0.5

Figure S4. Chemical composition analysis of *Ellerbeckia* in the region of valvocopula. (a) TEM image of part of the valvocopula, (b) elemental mapping of the area in red rectangle of (a), (c) element contents in this upper part. Pt and Ga content arises from the FIB preparation. Scale bar in (b): 0.5 μm.

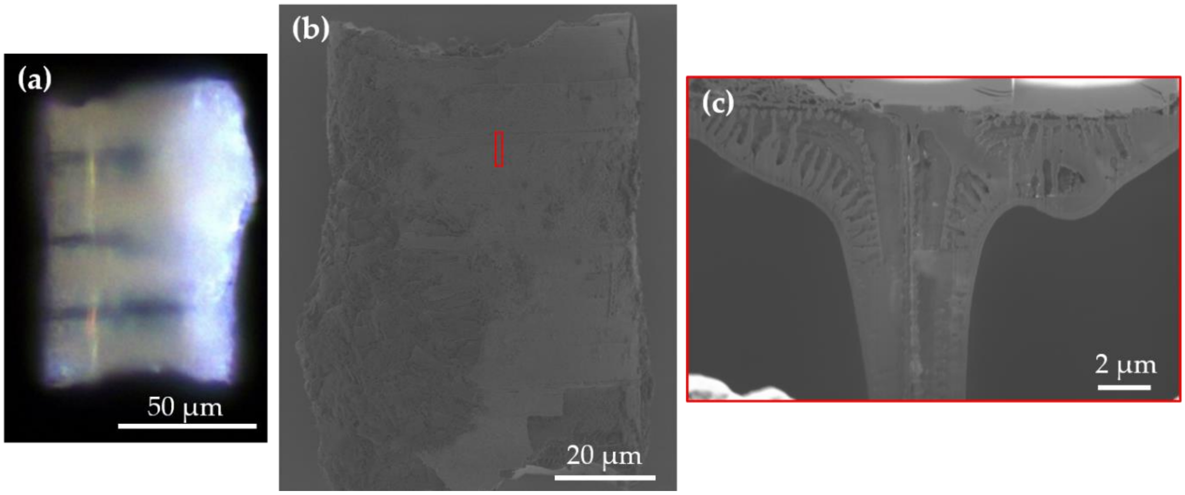

Figure S5. LM and SEM imaging of the *Melosira* frustule. (a) LM imaging of the *Melosira* frustule, (b and c) the same frustule from (a) prepared for the TEM lamella of the linking region of two valve faces.

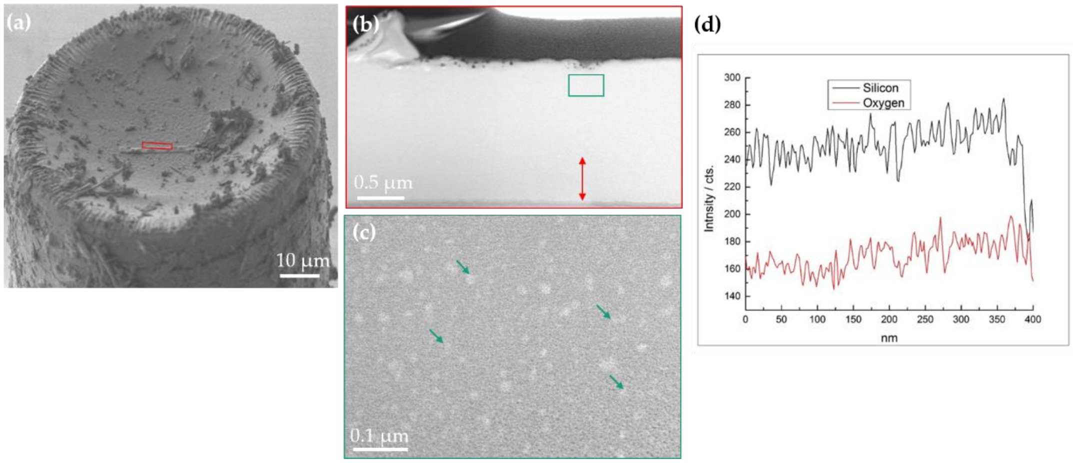

Figure S6. TEM imaging and EDX of *Ellerbeckia* in the center of the intaglio valve face. (a) SEM image of the *Ellerbeckia* intaglio valve face, (b) TEM imaging of the red area in (a), (c) high magnification image of the green area in (b), (d) line spectrum of elements in red double arrows of (b).

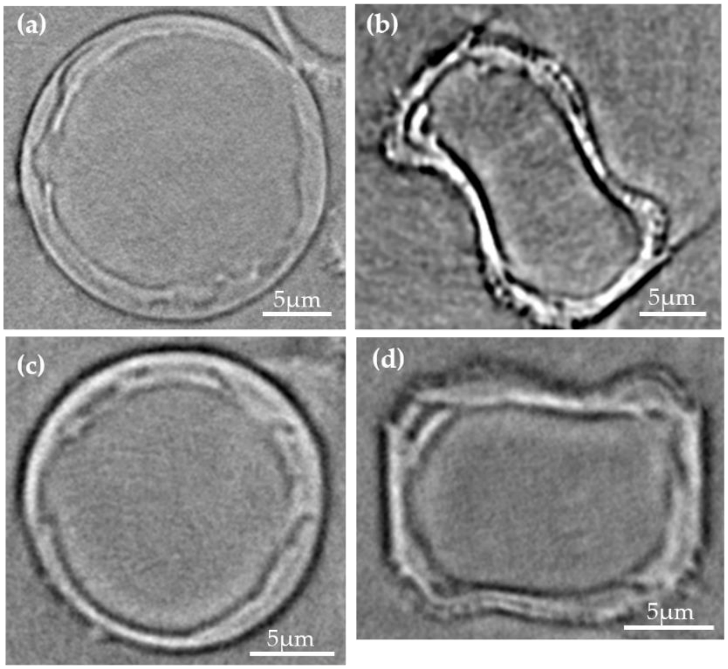

Figure S7. Nano-XCT images of *Thalassiosira lacustris* in valve view (a, c) and in girdle view (b, d) with valve diameter of 24.5 μm (a, b) and 17.7 μm (c, d), respectively.

Table S1. Structure parameters of the frustules from *Thalassiosira lacustris*

sample	Valve diameter (CD)	Valve thickness (VT)	Mantle wall thickness (MWT)	Copula thickness (CT)
	μm	μm	μm	μm
Thalassiosira lacustris 01	24.5 ± 0.1	1.21 ± 0.08	1.3 ± 0.1	0.95 ± 0.03
Thalassiosira lacustris 02	17.7 ± 0.4	1.20 ± 0.09	1.18 ± 0.08	0.97 ± 0.11
